# CEUS/CT fusion navigation for detection and interventional drainage of hidden peripancreatic abscesses in acute pancreatitis: a case report

**DOI:** 10.3389/fmed.2025.1520123

**Published:** 2025-07-09

**Authors:** Yuquan Wu, Ruizhi Gao, Jinshu Pang, Yun He, Hong Yang

**Affiliations:** Department of Medical Ultrasound, The First Affiliated Hospital of Guangxi Medical University, Nanning, China

**Keywords:** CEUS/CT fusion, virtual fusion navigation, peripancreatic abscess, acute pancreatitis, interventional guidance

## Abstract

**Background and aims:**

Multimodal imaging virtual fusion navigation offers higher accuracy and real-time guidance compared to single imaging modalities. This case report explores the value of contrast-enhanced ultrasound (CEUS)/CT fusion navigation in the localization, characterization, and interventional guidance of a hidden peripancreatic abscess in acute pancreatitis.

**Methods:**

The case involved the use of contrast-enhanced ultrasound and CT virtual fusion navigation to identify and assess the hidden peripancreatic abscess in a patient diagnosed with acute pancreatitis. The fusion technology allowed for real-time navigation during interventional procedures, offering improved lesion localization and characterization.

**Results:**

CEUS/CT fusion navigation successfully localized and characterized the hidden peripancreatic abscess. The real-time navigation provided by the fusion technology improved the accuracy and safety of the interventional procedure.

**Conclusion:**

CEUS/CT fusion navigation technology demonstrated significant value in the precise localization, detailed characterization, and effective interventional guidance of a hidden peripancreatic abscess in acute pancreatitis. This approach offers a promising tool for managing similar complex cases in clinical practice.

## Background

Acute pancreatitis is a common and potentially fatal disease, with up to one-fifth of patients developing pancreatic parenchymal necrosis, which can lead to sepsis and multiple organ failure (1, 2). Peripancreatic abscesses, common complications of acute pancreatitis, refer to the accumulation of infected pus in the peripancreatic tissue, further exacerbating the condition. Clinical management typically involves interventional or surgical treatment, making it crucial to confirm the presence and anatomical structure of the abscess. Ultrasound (US), computed tomography (CT), and magnetic resonance imaging (MRI) all exhibit high sensitivity and specificity for detecting peripancreatic abscesses (3). However, US can be limited by abdominal gas, potentially missing hidden or complex peripancreatic abscesses. CT and MRI sometimes struggle to differentiate between infected pus and inflammatory tissue, particularly when the abscess is small or in a concealed location. Therefore, exploring new methods for the localization and characterization of hidden or complex peripancreatic abscesses is of paramount importance.

## Technical description

Contrast-enhanced ultrasound (CEUS) is increasingly utilized in the diagnosis of tumors and diseases, particularly in liver diseases. Traditional intravenous CEUS has significantly improved the accuracy and sensitivity of liver tumor diagnosis (4, 5). With advancements in technology, the application value of multimodal imaging fusion techniques in diseases and tumors has become increasingly apparent. By integrating different imaging modalities, these techniques can provide more comprehensive lesion information and enhance diagnostic and therapeutic precision (6, 7). For instance, virtual fusion navigation technology has demonstrated excellent performance in the localization and treatment of liver tumors, precisely guiding biopsy and ablation procedures (8). For lesions that are visible on CT or MR but not clearly on US, US-CT/MR fusion imaging can be employed for navigational examination. Building on this, CEUS-CT/MR fusion imaging navigation can yield even more satisfactory results (9).

From a technical perspective, most peripancreatic abscesses are readily detectable on conventional US; however, when these abscesses are located deeper or covered by the intestines, US may not provide a clear view. Drawing on the successful experience of US/CT fusion imaging in the examination of liver lesions, CEUS/CT fusion imaging can be applied for the localization and characterization of peripancreatic abscesses. The specific procedure is as follows: first, the CT images that most clearly show the peripancreatic abscess are downloaded in DICOM format and imported into the US device for anatomical registration. Then, a real-time US examination is performed, with the lesion simultaneously observed on the CT images. SonoVue at a concentration of 59 mg per vial is dissolved in 5 mL of 0.9% saline, and 2.4 mL is injected via peripheral intravenous administration after thorough shaking. The location and anatomy of the peripancreatic abscess can be confirmed by identifying the non-enhanced area on CEUS, corresponding to the lesion shown on CT.

## Case description

**Figure 1:**
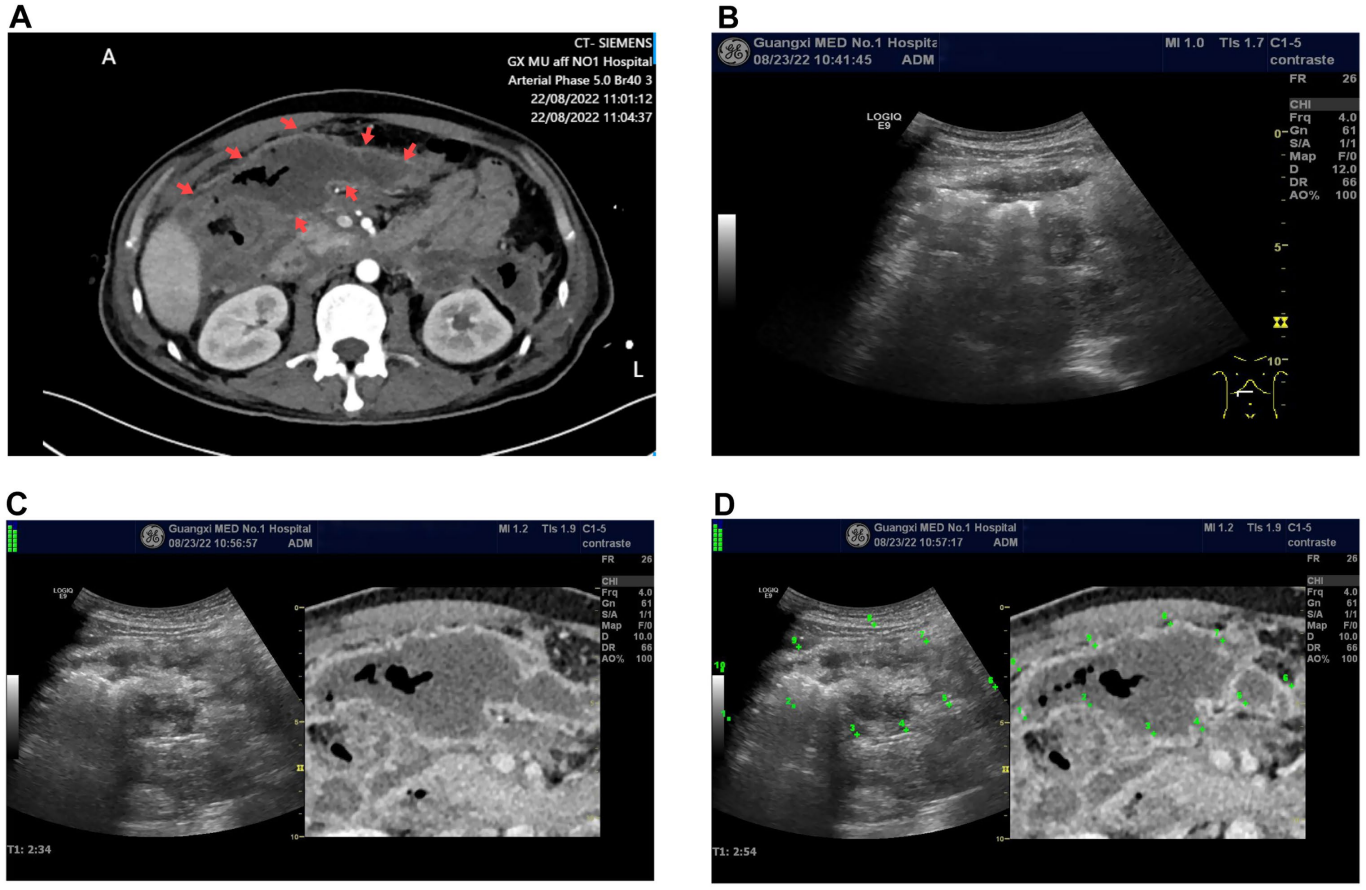
**(A)** Enhanced CT image of a peripancreatic abscess, where the target lesion appears as a low-density area (indicated by the arrow). It shows no enhancement on the enhanced CT, with gas density shadows inside. **(B)**. The grayscale ultrasound image at the corresponding location of the peripancreatic abscess indicated in the enhanced CT fails to distinguish the lesion. **(C)** The ultrasound-CT fusion imaging virtual navigation shows the peripancreatic abscess (unmarked), with the grayscale ultrasound image on the left and the enhanced CT image on the right. **(D)**. Point-to-point marking of the peripancreatic abscess as shown in **(C)**, using the enhanced CT delineation of the lesion as a reference, allows the corresponding lesion location to be observed on the ultrasound image.

The accompanying video presents a case of using CEUS/CT fusion imaging to diagnose a hidden peripancreatic abscess. The patient is a 37-year-old male who experienced upper abdominal pain and fever following heavy alcohol consumption 6 weeks prior. Laboratory tests revealed elevated white blood cell count, increased serum amylase, lipase, and urinary amylase levels, leading to a diagnosis of acute pancreatitis. Despite improvement following medication and peritoneal fluid drainage, the symptoms did not fully resolve. Bacterial culture of the peritoneal drainage fluid indicated a positive result for *Pseudomonas aeruginosa*. The latest CT scan still showed a peripancreatic abscess, necessitating a planned US-guided drainage procedure (Figure 1).

**Figure 2:**
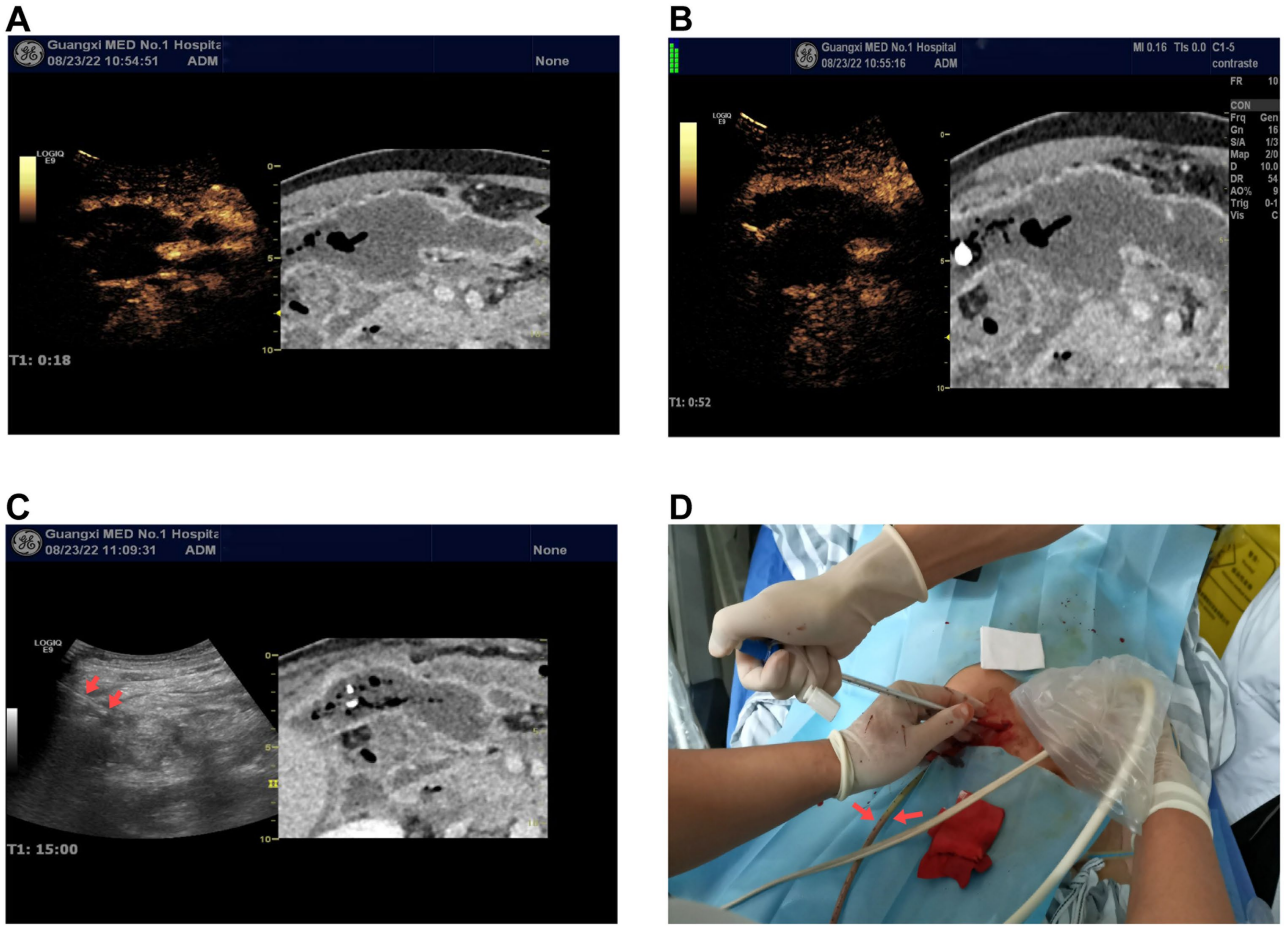
**(A,B)** Ultrasound contrast-enhanced CT fusion imaging virtual navigation shows early enhancement (18 s) and late enhancement (60s) images of the peripancreatic abscess. The left side of the images shows the ultrasound contrast-enhanced images, while the right side shows the enhanced CT images. Both the ultrasound contrast and enhanced CT images show the peripancreatic abscess as non-enhancing. **(C)** Percutaneous catheter drainage under ultrasound-CT fusion imaging virtual navigation. The arrow on the left ultrasound image indicates one of the catheters. **(D)** Intraoperative image of catheter placement as shown in **(C)**, demonstrating purulent fluid draining from one of the catheters.

The US equipment used was a GE LOGIQ E9, produced by General Electric (USA), equipped with a 3–5 MHz US probe and contrast-enhanced software. The procedure was performed by a physician with over 10 years of CEUS experience (Dr. Wu YQ). Conventional US examination failed to detect the peripancreatic abscess shown on CT. Following US/CT fusion imaging, the abscess was still not visible on the US images. Subsequently, CEUS/CT fusion imaging navigation was performed, revealing the peripancreatic abscess as a non-enhanced area on the CEUS image, corresponding to the abscess observed on CT (Supplementary Video 1). Using contrast-enhanced ultrasound/CT fusion imaging guidance, a Lingjie K-type 26F drainage tube was successfully placed via a one-step technique, yielding yellowish-green purulent fluid (Figure 2).

**Figure 3:**
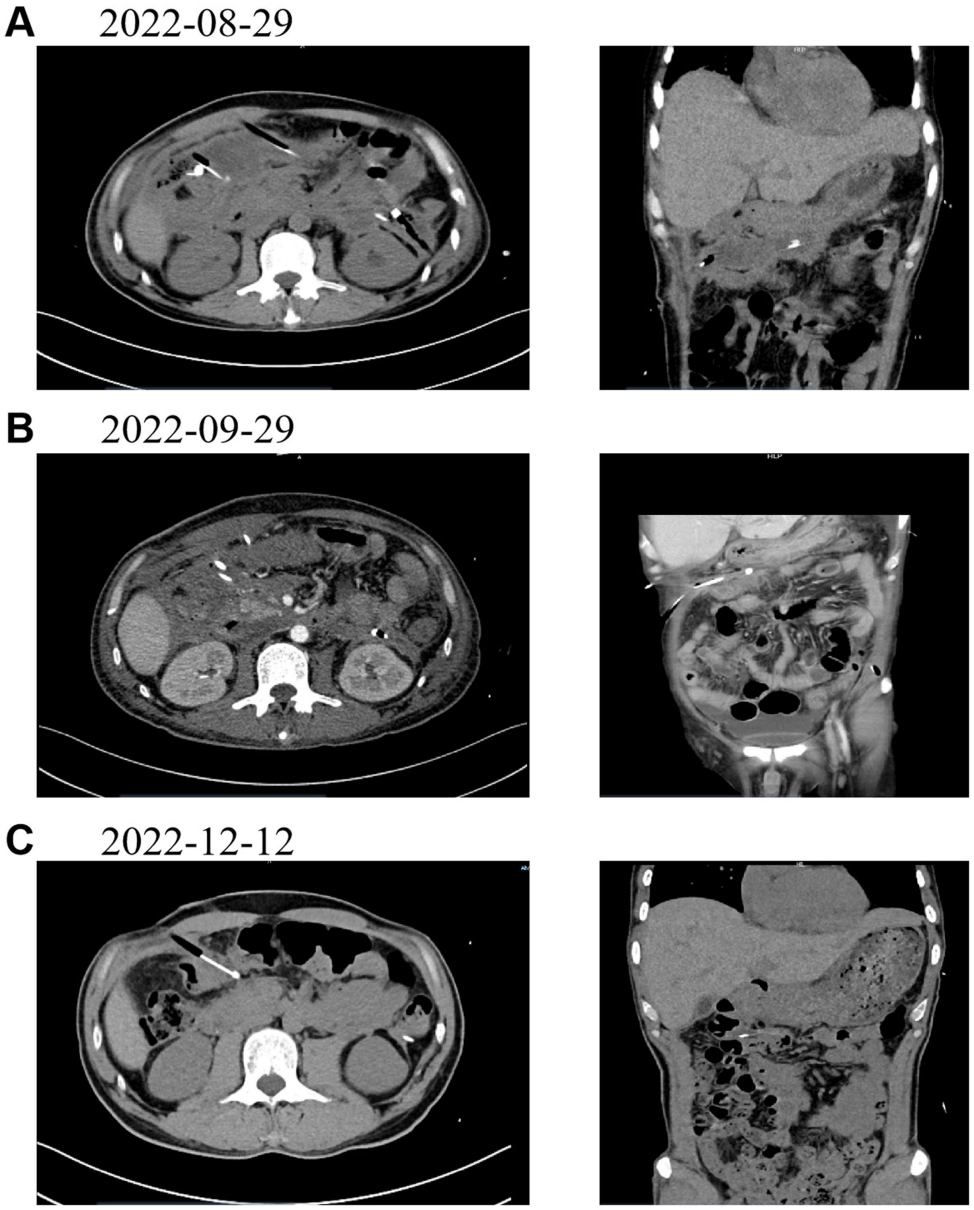
presents follow - up CT images of the patient at various time points post - drainage. **(A)** (August 29, 2022): CT image taken 1 week after the operation. **(B)** (September 29, 2022): CT image taken 1 month after the operation. **(C)** (December 12, 2022): CT image taken 4 months after the operation.

Postoperatively, antimicrobial therapy was initiated with piperacillin-tazobactam combined with amikacin based on ascitic fluid culture and antibiotic susceptibility testing results; however, due to inadequate clinical response, the treatment regimen was subsequently escalated to meropenem. Daily drainage output during the first five postoperative days measured 110 mL, 80 mL, 80 mL, 70 mL, and 70 mL respectively, demonstrating a consistent decreasing trend. Serial CT examinations performed at 1 week, 1 month, and 4 months post-intervention documented progressive reduction of peri-drainage fluid accumulation and gradual decrease in abscess dimensions, confirming effective drainage and sustained clinical improvement. At the most recent follow-up, there has been no radiological evidence of abscess recurrence and the patient remains asymptomatic, indicating favorable postoperative recovery. Corresponding CT images are provided as supplementary documentation to substantiate the clinical value of contrast-enhanced ultrasonography/CT fusion imaging in the management of such cases (see Figure 3).

## Conclusion

This case explores the practicality of CEUS/CT fusion imaging in detecting hidden peripancreatic abscesses. CEUS/CT fusion imaging can directly and clearly display the location and extent of peripancreatic abscesses, corresponding to the lesions shown on contrast-enhanced CT.

Conventional US was unable to display the peripancreatic abscess, likely due to the influence of intestinal coverage or the inability to distinguish heterogeneous echoes within the lesion from the intestines. Contrast-enhanced CT, unaffected by intestinal factors, can clearly display the lesion. CEUS/CT fusion imaging can detect hidden peripancreatic abscesses that conventional US cannot display, demonstrating significant clinical application and promotion value.

## Data Availability

The original contributions presented in the study are included in the article/Supplementary material, further inquiries can be directed to the corresponding author.

## Supplementary material

SUPPLEMENTARY VIDEO 1 Ultrasound contrast-enhanced CT fusion imaging shows the peripancreatic abscess. The peripancreatic abscess appears as non-enhancing on the ultrasound contrast (left side of the image) and as a low-density, non-enhancing lesion on the enhanced CT.
